# Pyrogallol induces oxidative stress defects in the fission yeast *S. pombe*

**DOI:** 10.17912/micropub.biology.000348

**Published:** 2021-01-07

**Authors:** Nafees Ahamad, Simmi Anjum, Shakil Ahmed

**Affiliations:** 1 Molecular and Structural Biology Division, CSIR- Central Drug Research Institute, Sector 10, Jankipuram Extension, Sitapur Road, Lucknow- 226031. India

## Abstract

Apart from the beneficial roles of pyrogallol in industries, it also tends to produce free radicals that trigger apoptosis in human cells. In this study, we checked the toxic effect of pyrogallol in fission yeast *S. pombe* cells. We observed that the wild type and *wat1/pop3* delete cells were unable to grow on plates containing pyrogallol in a dose-dependent manner. Furthermore, the *wat1/pop3* delete cells exhibit higher sensitivity against pyrogallol as compared to wild type cells suggesting that the pyrogallol induces oxidative stress. The exposure to pyrogallol also leads to the production of ROS and affects the sporulation in *S. pombe.*

**Figure 1:**
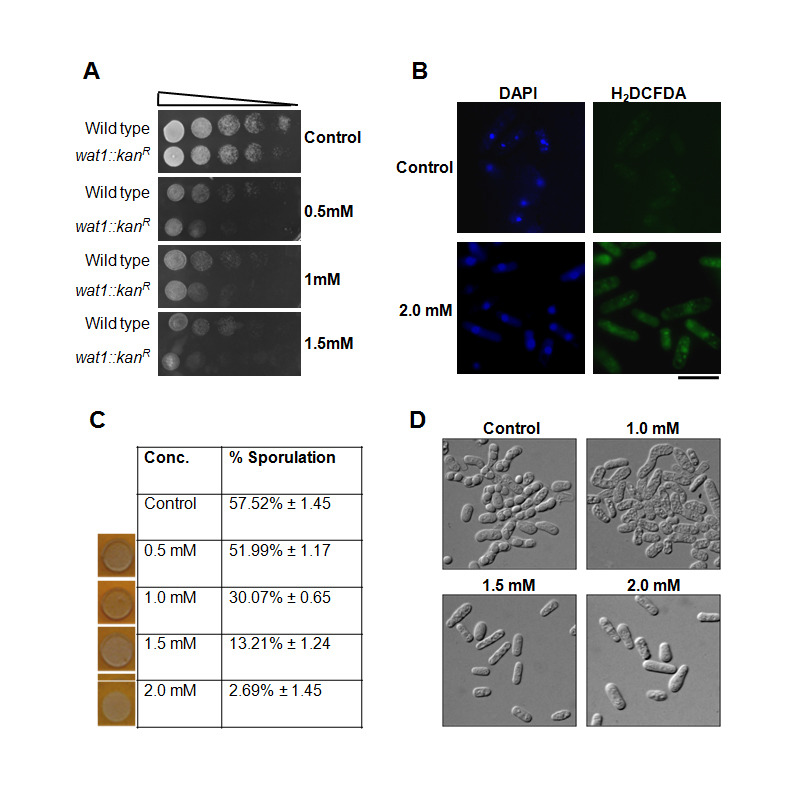
**(A)** Pyrogallol inhibits the growth of *S. pombe* cells: Wild type and *wat1/pop3* delete cells were grown till mid-log phase, 10-fold serial dilutions were spotted on plates containing indicated dose of pyrogallol. (**B)** Pyrogallol exposure leads to increased production of ROS: Wild type yeast cells were grown till mid-log phase, cells were treated with 2mM of pyrogallol at 30^o^C for 3 hrs. H_2_DCFDA dye was added and cells were further incubated for 90 min at 25^o^C to detect ROS. Scale bar, 10µm. (**C)** Pyrogallol treatment leads to sporulation defects: An equal number of h^+^ and h^–^ wild type yeast cells were mixed and spotted on malt extract plates containing the indicated dose of pyrogallol and plates were incubated at 30^o^C for 48hr. The left panel shows the colonies having spores stain dark after exposure with iodine vapour. The sporulation was also monitored under a microscope, approximately 100 cells inthree different experiments were examined for the asci formation and percent sporulation was calculated. **(D)** Photographic images of cells showing sporulation under different concentrations of pyrogallol.

## Description

Pyrogallol [C_6_H_3 _(OH)_3_] is an organic white water-soluble solid compound that finds several uses. It is used as an ingredient in the hair cosmetic industry (Mazzei *et al.*, 2007), photography (Upadhyay *et al.*, 2010), and as an antipsoriatic drug (Khan *et al.*, 2002). Besides its beneficiary roles in industries and as a consumer product, some previous studies report the toxicity caused by pyrogallol due to its tendency to produce free radicals and trigger apoptosis (Han and Park, 2002). Oxidative stress is mainly the cause of its toxicity and was reported to be carcinogenic and tumorigenic in female and male mice respectively (Mercado *et al.*, 2013). Humans are often exposed to pyrogallol through hair dyes and ingestion of beverages like tea, coffee, and cause deleterious effects if consumed beyond the permissible amount. However, some previous studies also suggest its therapeutic role in inhibiting tumor growth in certain animal models as well as induces G2-M arrest in human lung cancer cells (Yang *et al.*, 2009). Additionally, the protective effect of pyrogallol along with other phenols against the oxidative stress caused due to hydrogen peroxide has been reported in *Saccharomyces cerevisiae (Mendes *et al.*, 2015)*.

By considering these facts and background information, we examine the effects of pyrogallol treatment in fission yeast. We tested the dose-dependent consequences of pyrogallol on cell viability, sporulation efficacy, and intracellular ROS generation. The toxicity and cell viability parameters were checked in *S. pombe* at various concentrations by spot assay. We observed that the wild type cells were unable to grow on plates containing pyrogallol in a dose-dependent manner (Fig.1A). Previously, we have shown that the fission yeast *wat1/pop3* is required for the oxidative stress response and exhibits sensitivity towards hydrogen peroxide (Ahamad *et al.* 2016). To further ascertain the role of pyrogallol in oxidative stress response, we checked the pyrogallol sensitivity of *wat1/pop3* delete cells. The spotting assay revealed the enhanced pyrogallol sensitivity of *wat1/pop3* delete cells as compared to wild type cells (Fig. 1A) suggesting that the pyrogallol induces oxidative stress. Next, we examined the level of reactive oxygen species (ROS) in untreated control and pyrogallol treated wild type cells by staining with H_2_DCFDA dye which produces green fluorescence in the presence of ROS. We noticed a significant enhancement in green fluorescence under the confocal microscope displayed by pyrogallol treated cells in comparison with control (Fig. 1B) suggesting that the exposure to pyrogallol leads to increased production of ROS. Moreover, it has been reported from the previous studies that at hoisted concentrations, ROS deploys diverse detrimental effects on normal cellular pathways (Azad *et al.*, 2014). Previously we have shown that the increase in ROS due to the inactivation of Wat1/pop3 protein leads to defects in sporulation in fission yeast (Ahamad *et al.*, 2016; Ahamad *et al.*, 2018). To examine the effect of pyrogallol on the sporulation in *S. pombe* we mixed the wild type cells of opposite mating type on the minimal media plates containing various concentrations of pyrogallol. On control plates, nearly 57.5% asci with four spores were observed indicating the normal sporulation. In contrast, we observed a decrease in the number of asci on the plate containing pyrogallol in a dose-dependent manner. At 1mM and 2mM pyrogallol concentration, the sporulation was reduced to 30% and 2.7% respectively (Fig. 1C & D). Thus, our study demonstrates that oxidative stress is implicated in pyrogallol-mediated toxicity that leads to affect the sporulation in fission yeast *Schizosaccharomyces pombe*.

Taken together, the present study highlighted the role of pyrogallol inducing oxidative stress response in *S. pombe*. Alterations in the level of ROS caused by pyrogallol are one of its inherent pro-oxidant properties which cause cell death and sporulation defects. Our results emphasize that it is essential to verify the effects of pyrogallol through various modes to fully understand its mechanism. Hence, further studies are needed to identify the cellular and intracellular targets of this compound.

## Methods

**Growth sensitivity assay:** For the spotting experiment, wild type and *wat1/pop3* delete cells were grown at 25^o^C up to mid-log phase, 10^7^ cells were serially diluted, spotted on plates containing 0.5mM, 1mM, and 1.5mM pyrogallol. Plates were incubated at 25^o^C for 3-4 days before taking photograph.

**Intracellular ROS detection:** Wild type yeast cells were grown till mid-log phase in liquid YEA media and treated with 2mM of pyrogallol for approximately 3 hours. H_2_DCFDA dye was added and cells were further incubated for 90 min at 30^o^C to detect ROS by confocal microscope.

**Yeast sporulation assay:** *S. pombe* crosses were set up by mixing two strains of opposite mating types on malt extract agar media containing different doses of pyrogallol. The plates were incubated for 3 days at 30^o^C. The sporulation was monitored by staining the colonies with iodine vapour and also checked under the phase-contrast microscope.

## Reagents

**Reagents and yeast strains:** All the reagents unless specified or mentioned were purchased from HiMedia (India). Pyrogallol and H_2_DCFDA dye were obtained from Sigma-Aldrich. The stock solution of pyrogallol (10mM) was prepared in water. All the experiments were performed using the haploid strains of *S. pombe* (SP3: *h^+^leu1-32*; SH573: *h^+^ leu1-32 ura4D18 wat1::kan^R^*).

## References

[R1] Ahamad N, Sharma T, Khan S, Siddiqi MI, Ahmed S (2018). Phosphorylation of Wat1, human Lst8 homolog is critical for the regulation of TORC2 -Gad8 dependent pathway in fission yeast Schizosacchromyces pombe.. Eur J Cell Biol.

[R2] Ahamad N, Verma SK, Ahmed S (2016). Activation of Checkpoint Kinase Chk1 by Reactive Oxygen Species Resulting from Disruption of wat1/pop3 in Schizosaccharomyces pombe.. Genetics.

[R3] Azad GK, Singh V, Mandal P, Singh P, Golla U, Baranwal S, Chauhan S, Tomar RS (2014). Ebselen induces reactive oxygen species (ROS)-mediated cytotoxicity in Saccharomyces cerevisiae with inhibition of glutamate dehydrogenase being a target.. FEBS Open Bio.

[R4] Han YH, Park WH (2010). Pyrogallol-induced As4.1 juxtaglomerular cell death is attenuated by MAPK inhibitors via preventing GSH depletion.. Arch Toxicol.

[R5] Khan MT, Lampronti I, Martello D, Bianchi N, Jabbar S, Choudhuri MS, Datta BK, Gambari R (2002). Identification of pyrogallol as an antiproliferative compound present in extracts from the medicinal plant Emblica officinalis: effects on in vitro cell growth of human tumor cell lines.. Int J Oncol.

[R6] Mazzei JL, da Silva DN, Oliveira V, Hosomi RZ, do Val RR, Pestana CB, Felzenszwalb I (2006). Absence of mutagenicity of acid pyrogallol-containing hair gels.. Food Chem Toxicol.

[R7] Bartlik B, Galanter M, Angrist B (1989). Dimenhydrinate addiction in a schizophrenic woman.. J Clin Psychiatry.

[R8] Mercado-Feliciano M, Herbert RA, Wyde ME, Gerken DK, Hejtmancik MR, Hooth MJ (2012). Pyrogallol-associated dermal toxicity and carcinogenicity in F344/N rats and B6C3F1/N mice.. Cutan Ocul Toxicol.

[R9] Upadhyay G, Gupta SP, Prakash O, Singh MP (2009). Pyrogallol-mediated toxicity and natural antioxidants: triumphs and pitfalls of preclinical findings and their translational limitations.. Chem Biol Interact.

[R10] Yang CJ, Wang CS, Hung JY, Huang HW, Chia YC, Wang PH, Weng CF, Huang MS (2009). Pyrogallol induces G2-M arrest in human lung cancer cells and inhibits tumor growth in an animal model.. Lung Cancer.

